# Atrial Fibrillation, Cerebral Small Vessel Disease and Gender Medicine: Focus on Biomarkers and Neuroimaging

**DOI:** 10.3390/jcm15124427

**Published:** 2026-06-08

**Authors:** Francesco Alfano, Martina Berteotti, Francesca Cesari, Anna Maria Gori, Emilia Salvadori, Betti Giusti, Alessia Bertelli, Luca Bicchi, Filippo Fratini, Benedetta Formelli, Eleonora Barucci, Giulia Salti, Enrico Fainardi, Andrea Ginestroni, Stefano Chiti, Anna Poggesi, Rossella Marcucci

**Affiliations:** 1 Department of Experimental and Clinical Medicine, University of Florence, 50134 Florence, Italy; martina.berteotti@unifi.it (M.B.); francesca.cesari@gmail.com (F.C.); annamaria.gori@unifi.it (A.M.G.); betti.giusti@unifi.it (B.G.); alessia.bertelli@unifi.it (A.B.); bicchi.luca@gmail.com (L.B.); rossella.marcucci@unifi.it (R.M.); 2 Center for Atherothrombotic Diseases, Careggi University Hospital, 50134 Florence, Italy; 3 Department of Biomedical and Clinical Sciences, University of Milan, 20157 Milan, Italy; emilia.salvadori@unimi.it; 4 NEUROFARBA Department, Neuroscience Section, University of Florence, 50134 Florence, Italy; filippo.fratini@unifi.it (F.F.); benedetta.formelli@unifi.it (B.F.); eleonora.barucci@unifi.it (E.B.); anna.poggesi@unifi.it (A.P.); 5 Stroke Unit, Careggi University Hospital, 50134 Florence, Italy; saltig@aou-careggi.toscana.it; 6 Neuroradiology Unit, Careggi University Hospital, Department of Experimental and Clinical Biomedical Sciences, University of Florence, 50134 Florence, Italy; henryfai@tin.it (E.F.); a.ginestroni@gmail.com (A.G.); 7 Health Physics Unit, Careggi University Hospital, 50134 Florence, Italy

**Keywords:** atrial fibrillation, cerebral small vessel disease, female sex, biomarkers, neuroimaging, lacunar infarcts, white matter hyperintensities, small vessel disease score (SVDs), cerebral microbleeds, basal ganglia enlarged perivascular spaces (bgEPVSs)

## Abstract

**Background/Objectives**: Atrial fibrillation (AF) is the most common supraventricular arrhythmia and one of the most commonly encountered heart conditions in clinical practice. Emerging evidence suggests a significant role of inflammation, endothelial disfunction and extracellular matrix (ECM) remodeling in the pathogenesis of AF. Population studies have also suggested an association between AF and cerebral small vessel disease (CSVD), with growing evidence indicating that the burden of certain markers of CSVD is greater in women. However, the association between female sex and CSVD remains poorly understood. The aim of this study was thus to investigate the role of female sex in the association between circulating biomarkers and the presence of CSVD in AF patients undergoing oral anticoagulant therapy. **Methods**: The Strat-AF study is an observational, prospective, single-center, hospital-based study enrolling elderly patients with AF. Results refer to 170 patients (59 women and 111 men). Recruited patients are evaluated by means of a comprehensive protocol, with clinical, cerebral magnetic resonance imaging (MRI) and circulating biomarker assessments. **Results**: From a multivariate logistic regression analysis adjusted for multiple confounders, independent predictors were: in women, elevated vWF levels for the presence of lacunar infarcts [OR 3.24 (1.23–8.55), *p* = 0.018], elevated MMP-12, TIMP-1, TIMP-2, and TIMP-4 levels for the presence of CMBs [OR 7.76 (1.60–37.69), *p* = 0.021; OR 1.90 (1.02–3.52), *p* = 0.042; OR 2.46 (1.27–4.80), *p* = 0.008; and OR 2.36 (1.12–4.95), *p* = 0.023, respectively], elevated IL-6 and MMP-2 levels for the presence of WMH [OR 10.65 (1.31–86.67), *p* = 0.027; OR 3.36 (1.23–9.15), *p* = 0.018, respectively] and elevated MMP-12 and TIMP-2 levels for the presence of bgEPVS [OR 2.57 (1.22–5.93), *p* = 0.027; OR 2.15 (1.03–4.53), *p* = 0.043, respectively]; and in men: elevated TIMP-1 levels for the presence of WMH [OR 2.10 (1.08–4.08), *p* = 0.030], elevated TIMP-1 levels for the presence of bgEPVS [OR 2.20 (1.11–4.38), *p* = 0.025] and elevated TIMP-1 levels for SVDs positivity [OR 7.25 (2.18–24.15), *p* = 0.001]. **Conclusions**: These results from the Strat-AF study demonstrated that a complete biohumoral and instrumental assessment can jointly identify female patients with AF at higher risk of CSVD. These findings pave the way for the implementation of clinical protocols incorporating brain MRI and circulating biomarkers as potential innovative tools for an increasingly refined—and sex-specific—stratification of cardiovascular risk in AF patients undergoing oral anticoagulant therapy.

## 1. Introduction

Atrial fibrillation (AF) is the most common sustained cardiac arrhythmia in clinical practice, conferring an increased risk of ischemic stroke due to cardioembolism. AF is also linked to a range of other thromboembolic outcomes, including subclinical cerebral damage (potentially leading to vascular dementia) and thromboembolism to every other organ, all of which contribute to the higher risk of mortality associated with AF [1]. Despite the widespread use of oral anticoagulants (OAC), which substantially mitigate thromboembolic risk, there remains a residual risk [2,3]. Patients with AF remain vulnerable to cerebral small vessel disease (CSVD), manifesting as neuroradiological signs of cerebral microangiopathy, such as white matter hyperintensities (WMH), lacunar infarcts, and cerebral microbleeds (CMBs), observed in up to 70% of cases on magnetic resonance imaging (MRI) [4,5]. These microangiopathic changes are independently associated with cognitive decline, dementia, and functional impairment, underscoring the need to identify modifiable risk factors beyond traditional stroke prevention [6,7,8,9,10].

Circulating biomarkers of inflammation and extracellular matrix (ECM) remodeling have emerged as promising predictors of cerebral microangiopathy in cardiovascular disease. Inflammation drives endothelial dysfunction and vascular permeability, while ECM remodeling contributes to vessel wall instability and perivascular fibrosis, processes central to the pathogenesis of microangiopathy [11,12,13,14]. In AF cohorts, elevated levels of these biomarkers correlate with WMH burden and incident microbleeds, even under OAC protection, suggesting ongoing subclinical vascular injury [15]. However, the interplay between these biomarkers and imaging endpoints remains incompletely understood, particularly in the context of patient-specific factors.

Sex differences are increasingly recognized in cerebrovascular disease [16], with females exhibiting a higher prevalence and severity of WMH and CMBs, potentially mediated by hormonal influences, genetic predispositions, and differential inflammatory responses. Postmenopausal women with AF display amplified inflammatory profiles and ECM dysregulation compared to males, yet few studies have explored sex as a modifier in biomarker-microangiopathy associations [17]. Furthermore, sex-related differences in MMP/TIMP regulation have been described, with sex hormones, particularly estrogens, influencing extracellular matrix turnover, inflammatory responses, and vascular remodeling [18]. This gap is critical, as OAC-treated AF populations are predominantly female and older, where sex-stratified insights could inform personalized risk stratification.

The present study builds upon previously published findings from the Strat-AF study cohort [19] by specifically addressing a research question that has not been investigated before, namely the potential sex-related differences in the association between circulating biomarkers of inflammation and ECM remodeling and neuroradiological markers of cerebral microangiopathy in patients with AF undergoing OAC therapy. While previous Strat-AF analyses evaluated the overall relationship between these biomarkers and cerebral microangiopathy, the current study provides a dedicated sex-specific assessment aimed at clarifying whether female sex modulates these associations. By leveraging a prospective cohort with comprehensive biomarker profiling and brain MRI evaluation, we sought to identify potential sex-dependent mechanisms underlying the persistent cerebrovascular vulnerability observed in women with AF despite OAC. The aim of the study was thus to investigate the role of female sex in the association between circulating biomarkers and the presence of CSVD in AF patients undergoing oral anticoagulant therapy.

## 2. Materials and Methods

### 2.1. Study Population

The Strat-AF study (*Stratifying cerebral bleeding risk in AF*) is an observational, prospective, hospital-based study enrolling patients ≥ 65 years with a diagnosis of AF, ongoing OAC with VKA or with DOACs and with no contraindications for MRI. All the enrolled patients were referred from the outpatient clinic of Atherothrombotic Disease Center of Careggi University Hospital, where they are followed for the management of OAC in primary or secondary prevention of thromboembolic events.

The Strat-AF study was conducted in accordance with the Declaration of Helsinki and ethical approval was obtained from the Ethics Committee of Careggi University Hospital on 14 March 2017 (project identification code 16RFAP). The start date of the study was 18 September 2017.

All participants provided their written informed consent for inclusion before their enrollment.

The study design has been described in a previous paper [20], as well as the methodology regarding the laboratory determinations and the neuroimaging assessment [19].

### 2.2. Laboratory Determinations

Whole venous blood was collected in tubes without anticoagulant and with citrated whole blood (3.2%, 0.109 M). Tubes without and with anticoagulant were centrifuged at room temperature at 1500 *g* for 15 min, and the supernatants were stored in aliquots at −80 °C until the measurement of blood biomarkers, which was performed six months after enrollment. Samples were analyzed in a unique central laboratory. Levels of different inflammatory markers [interleukin (IL)-4, IL-6, IL-8, IL-10, tumor necrosis factor alpha (TNF-alpha), chemokine (C-C motif) ligand 2 (CCL2) also referred to as monocyte chemoattractant protein 1, C-X-C motif chemokine ligand 10 (CXCL10) also known as interferon gammainduced protein 10, intercellular adhesion molecule-1 (ICAM-1), vascular cell adhesion protein 1 (VCAM-1) and vascular-endothelial growth factor (VEGF)] were determined on serum samples using a Bio-Plex suspension array system and R&D Kits (R&D System, Milan, Italy). Metalloproteinases (MMP-2, MMP-7, MMP-8, MMP-9, MMP-12), extracellular matrix metalloproteinase inducer (EMMPRIN) and tissue inhibitors of metalloproteinases (TIMP-1, TIMP-2, TIMP-3 and TIMP-4) were assessed in the same serum samples using a Bio-Plex suspension array system (Bio-Rad Laboratories Inc., Hercules, CA, USA) and R&D Kits (R&D System, Milan, Italy) according to the manufacturer’s instructions. The coefficients of variation for inflammatory markers, MMPs and TIMPs assays were <6%. As regards clotting parameters, the activity of VWF was determined on citrated plasmas by a latex particle-enhanced immunoturbidimetric assay (Werfen, Milan, Italy). PAI-1 Antigen levels were assessed on plasma samples by immunoenzymatic assay (Hyphen Biomed, Neuville-sur-Oise, France).

### 2.3. Neuroimaging Assessment

Brain MRI were performed on a 1.5 T MRI (Ingenia, Philips Healthcare, Best, The Netherlands). The MRI protocol included the following sequences: sagittal T1-weighted spin-echo [repetition time (TR) = 547 ms; echo time (TE) = 12 ms; slice thickness = 5 mm; interslice spacing = 0.5 mm; matrix size = 320 × 250; field of view (FOV) = 23 cm × 23 cm; number of signals averaged (NSA) = 1], coronal T2-weighted fast spin-echo (TR = 3347 ms; TE = 110 ms; slice thickness = 5 mm; interslice spacing = 0.5 mm; matrix size = 512 × 322; FOV = 22 cm × 22 cm; NSA = 2); axial fluid-attenuated inversion recovery (FLAIR) [TR = 11,000 ms; TE = 125 ms; inversion time (TI) = 2800 ms; slice thickness = 5 mm; interslice spacing = 0.5 mm; matrix size = 384 × 204; FOV = 23 cm × 23 cm; NSA = 2]; axial gradient-echo T2 × (GRE) [TR = 534 ms; TE = 23 ms; flip angle (FA) = 18; slice thickness = 5 mm; interslice spacing = 0.5 mm; matrix size = 256 × 185; FOV = 23 cm × 23 cm; NSA = 1]; axial diffusion-weighted imaging (DWI) (TR = 3891 ms; TE = 75 ms; slice thickness = 5 mm; interslice spacing = 0.5 mm; matrix size = 164 × 162; FOV = 23 cm × 23 cm; NSA = 2); and gradient-echo 3D T1-weighted (TR = 7.5 ms; TE = 3.4 ms; TI = 950, slice thickness = 1 mm; matrix size = 256 × 241; FOV = 25.6 cm × 25.6 cm; NSA = 1) followed by multiplanar reconstruction (MPR) in axial, coronal, and sagittal planes. MRI was performed within 3 weeks from the blood sample collection [median and interquartile range: 13 (7–21) days].

The brain imaging protocol was planned and set up by imaging personnel with different expertise and skills, as suggested by current guidelines [21]. Cerebral lesion burden was visually assessed by two trained and experienced raters using validated scales. In particular, each MRI scan was evaluated by an expert neuroradiologist and a stroke neurologist. Both the neuroradiologist and the neurologist were blinded to any clinical information of the patients and/or results of biomarkers investigated. Cerebrovascular lesion burden encompassed markers of small vessel disease (SVD) and large vessel disease and included the following markers:

- Non-lacunar infarcts: defined as cortical or subcortical (>15 mm in diameter) lesions in vascular territories, they were numerically rated on T1-weighted and T2-FLAIR sequences.
- SVD markers were selected and evaluated according to the STRIVE criteria [22], and included the following:
  ○ White matter hyperintensities (WMH), rated on axial FLAIR sequences using the modified Fazekas scale [23], which defines three different grades of deep WMH severity: mild (single lesions < 10 mm; areas of “grouped” lesions < 20 mm in any diameter), moderate (single hyperintense lesions between 10 and 20 mm; areas of “grouped” lesions ≥ 20 mm in any diameter; no more than “connecting bridges” between individual lesions), and severe (single lesions or confluent areas of hyperintensity ≥ 20 mm in any diameter).
  ○ Cerebral microbleeds (CMBs), rated on axial gradient-echo T2-weighted sequences according to the Microbleeds Anatomical Rating Scale (MARS) [24], which identifies “definite” microbleeds as small, rounded or circular, well-defined hypointense lesions within brain parenchyma with clear margins ranging from 2 to 10 mm in size and classified location as deep, infratentorial and lobar.
  ○ Lacunar infarcts: lacunes of presumed vascular origin were detected and counted on T1 and T2 FLAIR sequences; they are defined as small (3–15 mm in diameter), round or ovoid, subcortical, fluid-filled cavities, usually surrounded by a hyperintense rim.
  ○ Enlarged perivascular spaces (EPVS), defined as fluid-filled spaces following small vessels course, with a round shape < 3 mm of diameter at basal ganglia (bgEPVS) level, hypointense in axial T1 images and rated estimating their absolute number in 3 slices of that anatomical region on the more injured side, and then categorized in the five-level scale: 0 = none, 1 = 1–10, 2 = 11–20, 3 = 21–40, 4 = >40 PVS per region [25].
  ○ SVD score (range 0–4: 0 = no signs, 1 = 1 sign, 2 = 2 signs, 3 = 3 signs, 4 = ≥3 signs), which incorporates 4 established neuroimaging biomarkers of SVD and aims to capture the overall burden of cerebral SVD [26]. The score was calculated as follows: presence of ≥1 lacunes (+1 point); presence of ≥1 MBs (+1 point); moderate/abundant (grade 2–4) bgEPVS (+1 point); moderate to severe WMHs

### 2.4. Statistical Analysis

As the main explanatory variable, we used the baseline levels of inflammatory markers, MMPs and TIMPs, vWF and PAI-1 antigen levels. Differences in these biomarker values were analyzed in relation to demographic and clinical features and across subgroups of patients with different outcomes.

We used Pearson’s χ2 to test for significance while comparing binary variables and ANOVA or Kruskal–Wallis H Test for numeric variables as appropriate. Values are presented as median and interquartile range if they have a non-Gaussian distribution.

To analyze differences in biomarker levels, we chose the Mann–Whitney U Test because of relatively large statistical variations. The net effect of each biomarker’s baseline on outcomes was then estimated by a logistic regression model, including as covariates age, sex, CHA2DS2-VASc, HAS-BLED and type of anticoagulant.

We chose variables for the adjustment of the multivariate logistic regression analysis according to the significance in the univariate analysis. We added to the multivariate analyses model the variables CHA2DS2-VASc and HASBLED, as these variables are associated with SVD lesions (microbleeds, lacunar and non-lacunar infarcts and SVD score).

Multicollinearity was assessed using variance inflation factors (VIFs) and tolerance statistics before multivariable logistic regression analysis.

Multivariable logistic regression analyses were performed using a backward stepwise selection model, and *p*-values were adjusted for multiple comparisons using the Benjamini–Hochberg false discovery rate correction. Continuous biomarkers were standardized (z-scores) prior to multivariable logistic regression analysis, and odds ratios are reported per one standard deviation increase.

A significant level was defined as *p* < 0.05. All analyses were performed with SPSS 20.0 (SPSS Inc., Chicago, IL, USA) and Stata 13.0 (Lakeway Dr, College Station, TX, USA).

## 3. Results

**Figure 1:**
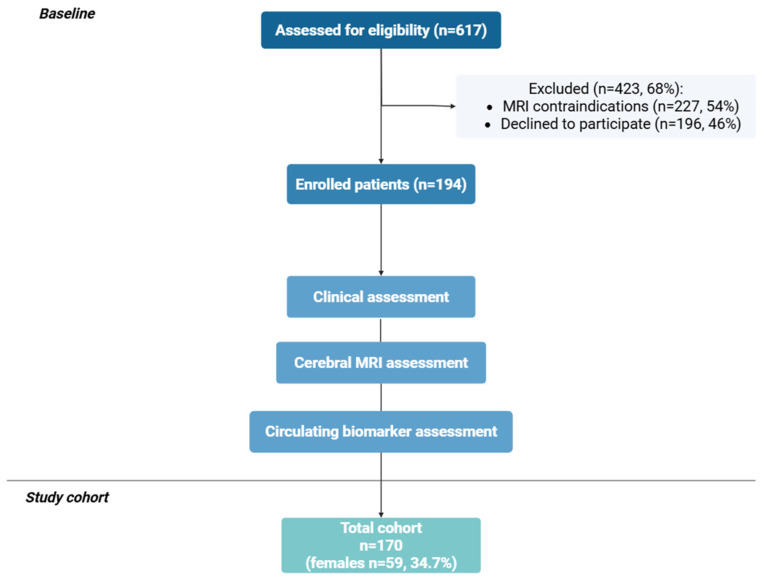
Strat-AF flow diagram (created by biorender.com).

Starting from September 2017 until March 2019, 617 patients referred to the outpatient clinic of the Center for Atherothrombotic Diseases for the management of OAC therapy were screened for inclusion in the study. As shown in Figure 1, 423 patients (68%) were excluded because of MRI contraindications (*n* = 227) or refusal (*n* = 196). The remaining 194 patients were enrolled. Out of the 194 subjects enrolled in the Strat-AF study, 170 completed the baseline MRI protocol and were included in the present study, so results refer to 170 subjects (mean age 77.7 ± 6.8 years, females *n* = 59, 34.7%).

Demographic and clinical characteristics are shown in Table 1. The subjects of the study were all on oral anticoagulant therapy: 30.6% (*n* = 52) were on VKA, whereas 69.4% (*n* = 118) were on DOACs. In particular, with regard to the DOACs group, 41 (34.7%) were on apixaban, 18 (15.3%) on edoxaban, 24 (20.3%) on rivaroxaban, and 35 (29.7%) on dabigatran; and in the VKA patients, 48 (92.3%) were treated with warfarin, whereas only 4 (7.7%) with acenocoumarol.

Regarding patients on VKAs, 81.3% (*n* = 39) of them had an adequate TTR (>60%). Concerning patients on DOACs, we assessed the anti-Xa activity using specific assays and found the majority of cases (>90%) to be in agreement with the trough levels reported in the literature (apixaban 22–177 ng/mL; dabigatran 61–143 ng/mL; edoxaban 19–62 ng/mL; rivaroxaban 6–239 ng/mL).

Subsequently, we performed a comparative analysis of the clinical and demographic characteristics based on sex (Table 2). All women recruited for the study were postmenopausal at the time of enrollment.

Women appeared to be less physically active than men (*p* = 0.006), but less exposed to smoke (*p* < 0.001) and alcohol consumption when compared to men (*p* = 0.001). Furthermore, men were more frequently affected by dyslipidaemia when compared to women (*p* = 0.028).

### 3.1. Circulating Biomarkers According to Cerebral Alterations by MRI Evaluation (Comparative Analysis Based on Sex)

#### 3.1.1. Lacunar and Non-Lacunar Infarcts

Women with lacunar infarcts had significantly higher levels of vWF with respect to women without lacunar infarcts (*p* = 0.004). On the other hand, men with lacunar infarcts had higher circulating levels of TIMP-1 when compared to men without lacunar infarcts (*p* = 0.009) (Table 3).

The analysis of the circulating biomarkers in relation to the presence of non-lacunar infarcts showed a significant increase in the circulating PAI-1 levels in women with non-lacunar infarcts (*p* = 0.020) and a significant increase in circulating levels of vWF and a significant reduction in circulating levels of MMP-9 in men with non-lacunar infarcts. (*p* = 0.011 and *p* = 0.040, respectively) (Table 4).

#### 3.1.2. Cerebral Microbleeds

Women with CMBs had higher circulating levels of IL-8 and MMP-12 (*p* = 0.008 and *p* = 0.026, respectively), as well as increased levels of TIMP-1, TIMP-2, TIMP-3, and TIMP-4 (*p* = 0.008, *p* = 0.002, *p* = 0.006, and *p* = 0.003, respectively) when compared to women without CMBs. On the other hand, men with CMBs had a significant increase in the circulating levels of MMP-2 when compared to men without CMBs (*p* = 0.013) (Table 5).

#### 3.1.3. White Matter Hyperintensity (WMH)

The analysis of the circulating biomarkers in relation to the presence of white matter hyperintensities (WMH) according to the Fazekas scale showed a significant increase in the circulating levels of MMP-2 and IL-6 in women with moderate-to-severe WMH when compared to women with absent or mild WMH (*p* = 0.022 and *p* = 0.042, respectively). In men, there was a significant increase in the circulating levels of MMP-2 and TIMP-1 in men with moderate-to-severe WMH when compared with men with absent or mild WMH (*p* = 0.012 and *p* = 0.023, respectively) (Table 6).

#### 3.1.4. Enlarged Perivascular Spaces Basal Ganglia (bgEPVS)

Women with bgEPVS showed a significant increase in the circulating levels of MMP-12 and TIMP-2 (*p* = 0.003 and *p* = 0.042, respectively), and men with bgEPVS had a significant increase in the circulating levels of IL-6 and TIMP-1 (*p* = 0.019 and *p* = 0.018, respectively) (Table 7).

#### 3.1.5. SVD Score

Women with at least one sign of CSVD according to the SVD score had higher circulating levels of vWF (*p* = 0.049), MMP-12 (*p* = 0.015), and of TIMP-1, TIMP-2, TIMP-3, and TIMP-4 (*p* = 0.011, *p* = 0.003, *p =* 0.032, and *p* = 0.009, respectively) with respect to women without signs of CSVD. Regarding men, there was a significant increase in the circulating levels of MMP-2 and TIMP-1 (*p* = 0.014 and *p* = 0.009, respectively) in men with evidence of at least one sign of CSVD when compared to patients without instrumental evidence of microangiopathy (Table 8).

### 3.2. Multivariate Analysis

In order to establish an independent association between the circulating biomarkers and the presence of brain MRI lesions in the two groups, we performed a multivariate logistic regression analysis adjusted for age, sex, CHA2DS2-VASc, HAS-BLED and type of anticoagulant.

**Figure 2:**
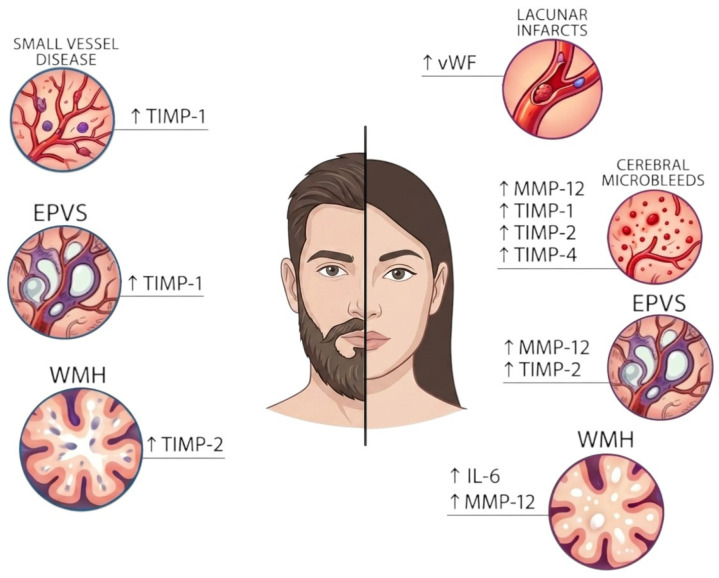
Independent predictors of clinical outcome in women and men in the Strat-AF Study population. Arrow: higher levels of the corresponding biomarker predict the presence of neuroimaging signs of cerebral small vessel disease (CSVD).

In the multivariate analysis, the following were identified as independent predictors of clinical outcome:

- Elevated vWF levels for the presence of lacunar infarcts in women [OR 3.24 (1.23–8.55), *p* = 0.018];
- Elevated MMP-12, TIMP-1, TIMP-2, and TIMP-4 levels for the presence of cerebral microbleeds (CMBs) in women [OR 7.76 (1.60–37.69), *p* = 0.021; OR 1.90 (1.02–3.52), *p* = 0.042; OR 2.46 (1.27–4.80), *p* = 0.008; and OR 2.36 (1.12–4.95), *p* = 0.023, respectively];
- Elevated IL-6 and MMP-2 levels for the presence of white matter hyperintensities in women [OR 10.65 (1.31–86.67), *p* = 0.027; OR 3.36 (1.23–9.15), *p* = 0.018, respectively];
- Elevated TIMP-1 levels for the presence of white matter hyperintensities in men [OR 2.10 (1.08–4.08), *p* = 0.030];
- Elevated MMP-12 and TIMP-2 levels for the presence of basal ganglia EPVS (bgEPVS) in women [OR 2.57 (1.22–5.93), *p* = 0.027; OR 2.15 (1.03–4.53), *p* = 0.043, respectively];
- Elevated TIMP-1 levels for the presence of bgEPVS in men [OR 2.20 (1.11–4.38), *p* = 0.025];
- Elevated TIMP-1 levels for SVD positivity in men [OR 7.25 (2.18–24.15), *p* = 0.001] (Figure 2).

## 4. Discussion

This work includes a part of the results of the Strat-AF study, which—among its multiple objectives—aimed to investigate the role of female sex in the association between selected circulating biomarkers of inflammation, endothelial dysfunction, and ECM remodeling and the presence of signs of CSVD, assessed by neuroimaging, in patients with AF receiving OAC.

To date, the pathogenesis of CSVD has not been fully elucidated. However, endothelial dysfunction and increased blood–brain barrier permeability have been implicated in its development. Experimental and neuroimaging studies have shown that systemic inflammatory markers and indicators of vascular inflammatory/endothelial dysfunction are associated with the prevalence and severity of CSVD.

At present, no studies have specifically investigated sex-related differences in the association between circulating biomarkers of inflammation, endothelial function, and extracellular matrix remodeling and imaging markers of CSVD. The only exception is a previous study conducted by our group, which assessed the predictive role of the same circulating biomarkers in identifying patients at higher risk of CMBs and CSVD, without specifically addressing the differential impact of sex on the development of those cerebral lesions [19].

In this context, these new findings represent a novel contribution to the medical literature and warrant further investigation to better elucidate the sex-specific pathophysiological mechanisms underlying CSVD lesion burden.

Specifically, our results showed that elevated vWF levels independently predicted the presence of lacunar infarcts in women. An association between vWF and lacunes has previously been reported in a Japanese study of 160 asymptomatic patients with imaging evidence of one or more lacunes. Patients were stratified into three subgroups according to the number of lacunes (1–2, 3–4, ≥5), and vWF levels were significantly higher in those with ≥5 lacunes compared with those with only 1–2 [27].

Our results regarding MMPs and TIMPs provide novel evidence supporting their potential involvement in the biological pathways associated with neuroimaging signs of CSVD in patients with AF. This association may be mediated by direct mechanisms, given the known involvement of MMPs in left atrial dilation, or through less specific pathways reflecting the overall burden of myocardial fibrosis. In healthy tissues, MMPs and TIMPs are maintained in a tightly regulated balance [28,29]; disruption of this equilibrium results in increased extracellular matrix degradation. Growing evidence supports the concept that MMPs and TIMPs operate within a positive feedback regulatory system: increased MMP activity alters the MMP/TIMP balance, triggering a compensatory upregulation of TIMPs. The association between MMPs, TIMPs, and neuroradiological markers of CSVD detected by magnetic resonance imaging (MRI) may, therefore, reflect MMP-mediated extracellular matrix degradation and inflammatory processes. Several clinical studies have demonstrated that metalloproteinases contribute to neurovascular matrix degradation and to the disruption of tight junction proteins and the cerebrovascular basal lamina, thereby promoting further brain injury [14,30].

Globally, CSVD represents a major subtype of vascular cognitive impairment, encompassing a spectrum of pathological processes affecting small arteries, arterioles, capillaries, and venules of the brain. It is one of the most common forms of cerebrovascular disease, particularly prevalent in the elderly population, and is associated not only with incident and recurrent stroke but also with cognitive decline and gait disturbances [31,32]. Diagnosis relies primarily on brain MRI, which allows identification of key imaging markers, including recent small subcortical infarcts, lacunes, white matter hyperintensities (WMH), basal ganglia enlarged perivascular spaces (bgEPVS), and cerebral microbleeds (CMBs).

From an epidemiological perspective, accumulating evidence suggests that the burden of certain CSVD markers, particularly WMH, is greater in women [33,34]. Female reproductive factors may influence late-life “brain trajectories,” potentially through the effects of estrogens on cerebrovascular function [35].

During the reproductive years, circulating estrogen levels fluctuate across the menstrual cycle and in response to major reproductive events. Cumulative lifetime hormonal exposure—determined by reproductive lifespan (from menarche to menopause) and number of pregnancies—may confer baseline protection that extends into the postmenopausal period [36]. Substantial evidence suggests that estrogens protect against endothelial dysfunction and atherosclerosis by promoting endothelial repair and angiogenesis, thereby reducing cardiovascular risk [37]. However, these protective effects diminish after menopause, when declining estrogen levels are associated with increased WMH burden compared with premenopausal women and age-matched men [38,39].

Overall, lifetime estrogen exposure is closely related to reproductive factors such as age at menarche, pregnancy history, and age at menopause, which may influence CSVD risk in women. Nevertheless, two randomized controlled trials failed to confirm the beneficial effects of daily estrogen therapy on brain structure [40,41]. Accordingly, the association between female reproductive characteristics and cSVD remains poorly defined, and current findings from epidemiological studies on this topic are still controversial.

The stronger association of TIMP-1 with WMH, bgEPVS and overall SVD burden in men may reflect sex-specific differences in extracellular matrix remodeling and vascular aging. TIMP-1 is a key regulator of matrix metalloproteinase activity, blood–brain barrier integrity, and vascular fibrosis. Previous studies have reported significant associations between circulating TIMP-1 levels and WMH severity in cerebral small vessel disease [42,43]. Therefore, TIMP-1–related pathways may contribute more prominently to cerebral microvascular injury in men, whereas in women, hormonal influences (particularly estrogen-mediated modulation of MMP/TIMP activity and vascular inflammation) may attenuate these relationships, leading to weaker associations between circulating TIMP-1 and SVD markers.

In our cohort, we were unable to correlate age at menarche, age at menopause, or pregnancy history with clinical endpoints, likely due to the relatively small number of women included in this unselected sample.

Our study has several limitations. First, the patient population was carefully selected according to the predefined inclusion criteria, and due to the relatively small number of events for certain outcomes, the multivariable models may have been affected by limited statistical power, unstable effect estimates, and potential overfitting, therefore limiting the generalizability of our findings. Larger studies are therefore needed to confirm and extend these results by increasing the statistical power and allowing for the detection of a more subtle relationship between variables. Second, the cross-sectional design precludes identification of patients at higher risk of subsequent thrombotic or hemorrhagic complications based on laboratory parameters, as the study provides a “snapshot” of the biomarker profile and MRI lesion burden at a single time point. Consequently, the results should be interpreted as hypothesis-generating rather than definitive evidence of a causal relationship between biomarkers and CSVD lesion development.

These findings from the Strat-AF study indicate that in a cohort of patients aged ≥65 years with AF receiving OAC for primary or secondary stroke prevention, there is a high prevalence of neuroradiological signs of CSVD. Cross-sectional biomarker assessment revealed enhanced activation of the endothelial pathways and ECM remodeling in patients with neuroimaging evidence of CSVD. Notably, several biomarkers (vWF, MMP-12, TIMP-1, TIMP-2, TIMP-4) appeared to disproportionately affect women, in whom these molecules were independently associated with a greater lesion burden compared to men (who were numerically more represented in the sample).

## 5. Conclusions

In conclusion, the Strat-AF study demonstrates that advanced neuroimaging assessment by brain MRI, combined with biomarker profiling of inflammatory, hemostatic, and endothelial activation pathways, may identify women with AF at increased risk of neuroradiological manifestations of CSVD. These findings highlight the potential clinical relevance of a multimodal and sex-specific approach to vascular risk assessment, supporting the use of brain MRI and circulating biomarkers for a more refined characterization of cerebrovascular vulnerability in selected patients with AF. While these results provide a strong rationale for incorporating such markers into future risk stratification strategies, confirmation in larger, multicenter, and longitudinal cohorts is required before routine clinical implementation. Future studies should determine whether the integration of neuroimaging and biomarker data into existing risk assessment models can improve individualized therapeutic decision-making and ultimately reduce cerebrovascular complications in patients with AF considered for OAC therapy.

## Figures and Tables

**Table 1:** Demographic and clinical characteristics of the baseline Strat-AF study cohort (*n* = 170). Results are expressed as median ± DS and as a percentage.

Demographic and Clinical Characteristics	Total Cohort (*n* = 170)
Age [years], (mean ± SD)	77.7 ± 6.8
Female sex, *n* (%)	59 (34.7%)
Schooling [years], (mean ± SD)	9.1 ± 4.3
Stroke, *n* (%)	38 (22.4%)
Coronary artery disease, *n* (%)	18 (10.6%)
Heart failure, *n* (%)	25 (14.7%)
Peripheral arterial disease, *n* (%)	14 (8%)
Hypertension, *n* (%)	140 (82.4%)
Diabetes, *n* (%)	22 (12.9%)
Dyslipidaemia, *n* (%)	87 (51.2%)
Physical activity (lack of), *n* (%)	110 (64.7%)
Smoke, *n* (%)	105 (61.8%)
Alcohol consumption, *n* (%)	91 (53.5%)
BMI [kg/m^2^], (mean ± SD)	26.3 ± 3.9
CHA_2_DS_2_-VASc Score (mean ± SD)	3.69 ± 1.49
HAS-BLED (mean ± SD)	1.85 ± 0.89

**Table 2:** Comparative analysis of the clinical and demographic characteristics based on sex of the baseline Strat-AF study cohort (*n* = 170). Results are expressed as median ± DS and as a percentage.

	Women (*n* = 59)		Men (*n* = 111)		*p*
Age [years], (mean ± SD)	78.5 ± 7.4		77.43 ± 6.36		0.333
**Schooling [years], (mean ± SD)**	**7.1 ± 3.4**		**10.55 ± 4.30**		**<0.001**
BMI [kg/m^2^], (mean ± SD)	26.4 ± 4.37		26.30 ± 3.60		0.804
**CHA_2_DS_2_-VASc (mean ± SD)**	**4.5 ± 1.5**		**3.3 ± 1.4**		**<0.001**
HAS-BLED (mean ± SD)	1.9 ± 0.9		1.8 ± 0.9		0.859
	**Women** **(*n* = 59)**		**Men** **(*n* = 111)**		***p***
	Yes	No	Yes	No	
Stroke, *n* (%)	18	41	20	91	0.063
Coronary artery disease, *n* (%)	3	56	15	96	0.089
Heart failure, *n* (%)	8	51	17	94	0.758
Peripheral arterial disease, *n* (%)	3	56	11	100	0.276
Hypertension, *n* (%)	49	10	91	20	0.862
Diabetes, *n* (%)	7	52	15	96	0.760
**Dyslipidaemia, *n* (%)**	**37**	**22**	**50**	**61**	**0.028**
**Physical activity (lack of), *n* (%)**	**12**	**47**	**48**	**63**	**0.003**
Smoke [current], *n* (%)	2	57	10	101	0.173
**Smoke [previous], *n* (%)**	**19**	**40**	**76**	**35**	**<0.001**
**Alcohol consumption, *n* (%)**	**21**	**38**	**70**	**41**	**<0.001**

**Table 3:** Circulating biomarkers in relation to the presence of lacunar infarcts in women and men. Results are expressed as median (range).

			IL-4 [pg/mL]	IL-6 [pg/mL]	IL-8 [pg/mL]	IL-10 [pg/mL]	TNF-α [pg/mL]	CCL-2 [pg/mL]	CXCL-10 [pg/mL]	ICAM-1 [ng/mL]	VCAM-1 [ng/mL]	VEGF [pg/mL]	PAI-1 [ng/mL]	vWF [%]	EMMPRIN [ng/mL]	MMP-2 [ng/mL]	MMP-7 [ng/mL]	MMP-8 [ng/mL]	MMP-9 [ng/mL]	MMP-12 [ng/mL]	TIMP-1 [ng/mL]	TIMP-2 [ng/mL]	TIMP-3 [ng/mL]	TIMP-4 [ng/mL]
**Lacunar infarcts**	**Women** (*n* = 59)	**Present** (*n* = 9)	12.81	2.73	12.06	0.92	2.04	330.89	14.91	380.37	1740.10	116.25	9.35	**217.80**	4.36	532.82	3.39	6.07	259.25	309.12	181.77	141.11	52.59	3.75
			(9.46–36.03)	(1.74–6.31)	(6.92–16.28)	(0.27–3.63)	(1.53–5.39)	(285.75–447.78)	(10.62–24.39)	(301.20–538.41)	(1456.05–1968.15)	(52.70–129.84)	(4.81–22.88)	**(179.65–234.25)**	(2.50–7.11)	(448.76–800.74)	(2.63–6.07)	(4.33–16.38)	(151.54–478.12)	(125.27–590.88)	(148.64–261.00)	(123.29–224.65)	(26.04–61.04)	(2.85–7.83)
		**Absent** (*n* = 50)	12.81	1.85	7.74	2.89	2.29	341.32	16.31	341.49	1398.30	76.48	9.29	**147.10**	5.78	494.95	4.80	7.20	301.61	450.10	159.15	123.38	32.53	3.54
			(5.30–26.75)	(0.38–3.58)	(4.65–11.58)	(0.41–3.56)	(1.01–4.00)	(232.29–492.44)	(9.97–21.16)	(273.07–415.38)	(950.72–2351.98)	(37.65–113.80)	(6.98–14.44)	**(127.60–200.05)**	(4.35–6.88)	(384.99–660.17)	(2.77–6.10)	(1.98–13.02)	(170.83–413.36)	(72.57–594.96)	(124.53–216.77)	(97.82–163.74)	(17.61–51.01)	(2.39–5.24)
		***p***	0.410	0.170	0.073	0.398	0.534	0.850	0.800	0.332	0.255	0.214	0.726	**0.004**	0.217	0.229	0.550	0.784	0.916	0.784	0.177	0.146	0.250	0.354
	**Men** (*n* = 111)	**Present** (*n* = 28)	6.60	1.35	9.66	2.89	2.02	332.46	14.00	329.80	1201.90	77.38	9.19	195.70	6.21	527.52	6.01	9.12	323.59	132.64	**187.26**	147.33	43.98	2.88
			(2.54–34.48)	(0.30–1.98)	(7.26–13.78)	(0.23–3.50)	(0.59–3.16)	(252.66–425.36)	(11.00–20.96)	(244.58–764.08)	(961.36–1828.50)	(44.22–110.42)	(6.71–15.76)	(130.05–217.23)	(4.40–7.58)	(459.95–625.43)	(4.35–7.13)	(4.11–17.68)	(185.72–572.08)	(50.50–487.40)	**(163.53–233.35)**	(119.28–200.90)	(30.52–65.07)	(2.33–6.32)
		**Absent** (*n* = 83)	10.81	1.56	8.14	3.20	2.30	308.67	14.34	319.52	1480.00	53.61	9.03	158.90	5.17	529.83	5.97	7.88	312.24	309.12	**157.40**	124.95	34.43	2.95
			(5.00–30.48)	(0.30–3.25)	(5.32–13.24)	(0.32–3.56)	(1.06–4.25)	(218.76–421.91)	(9.96–24.76)	(251.23–500.96)	(1010.00–2114.40)	(33.64–88.53)	(7.25–16.42)	(128.00–210.20)	(3.81–6.55)	(439.71–627.23)	(3.42–7.24)	(4.00–13.70)	(199.03–465.70)	(65.70–594.96)	**(129.82–198.55)**	(95.82–168.77)	(25.45–50.02)	(2.12–3.67)
		***p***	0.729	0.316	0.176	0.137	0.300	0.541	0.796	0.770	0.138	0.142	0.623	0.394	0.159	0.607	0.701	0.661	0.701	0.389	**0.009**	0.105	0.082	0.214

**Table 4:** Circulating biomarkers in relation to the presence of non-lacunar infarcts in women and men. Results are expressed as median (range).

			IL-4 [pg/mL]	IL-6 [pg/mL]	IL-8 [pg/mL]	IL-10 [pg/mL]	TNF-α [pg/mL]	CCL-2 [pg/mL]	CXCL-10 [pg/mL]	ICAM-1 [ng/mL]	VCAM-1 [ng/mL]	VEGF [pg/mL]	PAI-1 [ng/mL]	vWF [%]	EMMPRIN [ng/mL]	MMP-2 [ng/mL]	MMP-7 [ng/mL]	MMP-8 [ng/mL]	MMP-9 [ng/mL]	MMP-12 [ng/mL]	TIMP-1 [ng/mL]	TIMP-2 [ng/mL]	TIMP-3 [ng/mL]	TIMP-4 [ng/mL]
**Non lacunar infarcts**	**Women** (*n* = 59)	**Present** (*n* = 22)	12.81	1.71	5.99	2.50	2.29	319.06	16.35	338.70	1404.65	78.85	**12.40**	173.60	5.69	512.60	5.16	7.05	320.63	450.10	178.18	136.96	40.14	3.93
			(5.98–31.00)	(1.49–2.99)	(4.11–11.37)	(0.40–3.46)	(2.02–5.01)	(268.11–569.97)	(10.32–21.71)	(286.43–565.77)	(885.97–2255.53)	(46.33–120.26)	**(9.26–17.29)**	(124.45–201.80)	(3.78–7.46)	(412.47–653.96)	(2.63–6.54)	(1.98–12.43)	(162.33–423.14)	(81.34–594.96)	(152.05–259.75)	(104.10–220.94)	(26.80–67.36)	(2.53–6.12)
		**Absent** (*n* = 37)	12.81	2.21	8.69	3.00	2.00	345.99	15.77	342.99	1518.80	78.44	**8.37**	154.30	5.78	470.58	4.23	7.07	279.06	450.10	148.81	118.07	30.49	3.52
			(5.20–32.83)	(0.38–4.60)	(5.05–12.53)	(0.32–3.91)	(0.73–4.00)	(237.61–456.94)	(10.13–22.20)	(278.17–413.43)	(1050.95–2059.40)	(40.94–121.33)	**(6.46–11.13)**	(131.30–207.70)	(4.35–6.87)	(386.51–711.62)	(2.77–5.59)	(3.51–15.59)	(178.93–416.30)	(49.73–594.96)	(123.80–209.22)	(98.10–158.30)	(17.18–49.74)	(2.54–5.33)
		***p***	0.969	0.666	0.256	0.212	0.530	0.969	0.766	0.808	0.610	0.944	**0.020**	0.999	0.760	0.713	0.348	0.938	0.808	0.540	0.101	0.327	0.124	0.666
	**Men** (*n* = 111)	**Present** (*n* = 37)	13.80	1.49	8.51	3.21	2.03	309.83	15.04	318.96	1350.00	55.94	11.96	**197.30**	5.41	536.47	6.20	7.87	**264.77**	221.62	161.46	139.16	35.51	2.89
			(5.50–37.59)	(0.30–2.86)	(6.74–13.27)	(0.58–3.73)	(1.06–4.10)	(222.00–392.07)	(11.90–25.83)	(247.99–542.97)	(1036.05–2022.90)	(43.01–109.94)	(7.47–18.42)	**(155.30–221.70)**	(3.80–7.11)	(455.32–656.91)	(4.19–7.24)	(3.27–11.72)	**(177.62–369.81)**	(41.46–511.55)	(131.07–210.82)	(112.08–187.90)	(27.56–51.53)	(2.47–3.90)
		**Absent** (*n* = 74)	6.60	1.56	8.16	2.89	2.21	314.02	13.29	325.63	1364.50	59.97	8.61	**150.60**	5.32	523.07	5.90	8.35	**349.67**	309.12	167.64	125.51	36.11	2.97
			(4.93–29.67)	(0.38–2.95)	(4.26–13.54)	(0.30–3.56)	(0.65–4.17)	(221.11–443.07)	(9.70–21.30)	(250.93–559.72)	(995.59–1983.23)	(32.56–90.31)	(6.96–13.20)	**(120.40–204.60)**	(3.84–7.00)	(428.05–622.74)	(3.56–7.11)	(4.46–17.30)	**(204.19–557.44)**	(65.70–590.88)	(132.65–205.18)	(95.53–182.55)	(24.80–54.51)	(2.13–4.04)
		***p***	0.272	0.566	0.451	0.465	0.925	0.374	0.134	0.965	0.993	0.418	0.111	**0.011**	0.923	0.316	0.592	0.149	**0.040**	0.166	0.947	0.364	0.982	0.840

**Table 5:** Circulating biomarkers in relation to the presence of CMBs in women and men. Results are expressed as median (range).

			IL-4 [pg/mL]	IL-6 [pg/mL]	IL-8 [pg/mL]	IL-10 [pg/mL]	TNF-α [pg/mL]	CCL-2 [pg/mL]	CXCL-10 [pg/mL]	ICAM-1 [ng/mL]	VCAM-1 [ng/mL]	VEGF [pg/mL]	PAI-1 [ng/mL]	vWF [%]	EMMPRIN [ng/mL]	MMP-2 [ng/mL]	MMP-7 [ng/mL]	MMP-8 [ng/mL]	MMP-9 [ng/mL]	MMP-12 [ng/mL]	TIMP-1 [ng/mL]	TIMP-2 [ng/mL]	TIMP-3 [ng/mL]	TIMP-4 [ng/mL]
**CMBs**	**Women** (*n* = 59)	**Present** (*n* = 8)	29.4	2.73	**14.99**	0.95	3.03	345.13	11.67	345.96	1825.05	71.43	9.47	161.50	6.97	656.87	5.53	8.80	308.55	**594.96**	**248.23**	**223.73**	**61.78**	**6.30**
			(6.10–40.28)	(0.78–4.36)	**(9.08–18.55)**	(0.26–3.35)	(0.69–6.21)	(267.74–843.49)	(8.58–15.96)	(297.98–406.85)	(1071.80–2452.03)	(53.41–112.62)	(7.01–10.71)	(109.20–207.50)	(5.80–10.21)	(468.51–1041.50)	(3.51–6.31)	(3.00–17.07)	(133.45–392.61)	**(456.97–693.21)**	**(201.01–328.27)**	**(152.04–352.69)**	**(41.30–82.99)**	**(4.33–9.16)**
		**Absent** (*n* = 51)	12.81	1.85	**7.42**	2.89	2.04	338.48	16.37	341.75	1406.20	78.44	8.99	163.95	5.51	493.74	4.50	6.78	299.81	**414.58**	**154.55**	**118.07**	**31.92**	**3.36**
			(5.40–23.92)	(0.65–3.77)	**(4.65–11.58)**	(0.44–3.56)	(1.51–4.00)	(242.92–485.08)	(10.86–22.30)	(274.28–463.87)	(1006.50–2044.30)	(37.71–127.60)	(6.69–15.93)	(131.05–203.63)	(4.23–6.50)	(389.08–643.47)	(2.61–6.28)	(3.51–12.97)	(178.16–420.08)	**(46.35–586.80)**	**(125.26–202.86)**	**(98.05–146.91)**	**(18.04–50.02)**	**(2.40–4.66)**
		***p***	0.312	0.825	**0.008**	0.098	0.868	0.626	0.135	0.929	0.341	0.965	0.628	0.851	0.054	0.073	0.430	0.550	0.713	**0.026**	**0.008**	**0.002**	**0.006**	**0.003**
	**Men** (*n* =111)	**Present** (*n* = 21)	6.10	1.56	8.14	3.21	2.53	243.99	16.99	314.15	1249.50	75.34	10.63	159.80	5.89	**619.67**	6.04	6.99	295.33	309.12	189.57	148.67	38.82	3.18
			(3.77–29.94)	(0.34–1.94)	(6.59–13.20)	(0.32–3.99)	(0.59–3.83)	(206.76–382.26)	(12.21–25.20)	(243.82–703.03)	(1034.48–2022.90)	(35.15–120.13)	(7.05–13.29)	(129.70–217.30)	(3.18–7.64)	**(501.03–676.19)**	(2.99–8.10)	(3.40–13.66)	(204.09–513.40)	(49.73–594.96)	(128.97–217.29)	(111.06–201.02)	(28.13–63.08)	(1.86–5.60)
		**Absent** (*n* = 90)	11.81	1.50	8.64	2.92	2.04	314.02	13.54	323.63	1404.55	56.36	8.85	178.50	5.32	**511.15**	5.97	8.22	319.50	309.12	163.93	131.02	35.51	2.89
			(5.00–33.47)	(0.30–3.12)	(5.40–14.19)	(0.30–3.52)	(1.06–4.17)	(221.11–435.15)	(9.92–23.75)	(251.68–513.74)	(1002.63–1983.23)	(36.32–89.44)	(7.11–16.71)	(127.00–212.70)	(3.99–6.60)	**(433.51–596.67)**	(3.68–7.09)	(4.19–14.61)	(190.29–482.20)	(63.58–527.74)	(132.65–199.03)	(96.45–177.86)	(24.80–51.94)	(2.16–3.97)
		***p***	0.472	0.904	0.792	0.351	0.934	0.259	0.283	0.769	0.781	0.577	0.949	0.988	0.952	**0.013**	0.718	0.472	0.811	0.781	0.707	0.396	0.436	0.787

**Table 6:** Circulating biomarkers in relation to the presence of WMH (dichotomized Fazekas scale) in women and men. Results are expressed as median (range).

			IL-4 [pg/mL]	IL-6 [pg/mL]	IL-8 [pg/mL]	IL-10 [pg/mL]	TNF-α [pg/mL]	CCL-2 [pg/mL]	CXCL-10 [pg/mL]	ICAM-1 [ng/mL]	VCAM-1 [ng/mL]	VEGF [pg/mL]	PAI-1 [ng/mL]	vWF [%]	EMMPRIN [ng/mL]	MMP-2 [ng/mL]	MMP-7 [ng/mL]	MMP-8 [ng/mL]	MMP-9 [ng/mL]	MMP-12 [ng/mL]	TIMP-1 [ng/mL]	TIMP-2 [ng/mL]	TIMP-3 [ng/mL]	TIMP-4 [ng/mL]
**WMH**	**Women** (*n* = 59)	**Score 2–3** (*n* = 39)	12.81	**2.24**	5.97	3.00	2.53	328.41	16.31	355.66	1518.80	78.44	10.28	172.650	5.53	**532.82**	4.69	7.51	324.64	450.10	170.64	133.68	38.82	4.13
			(5.60–35.81)	**(1.49–5.17)**	(4.65–11.58)	(0.32–3.56)	(1.51–5.00)	(273.90–458.84)	(10.41–20.54)	(285.74–491.33)	(1113.50–2191.30)	(48.65–121.23)	(7.07–17.04)	(131.30–218.78)	(4.03–6.90)	**(448.32–730.37)**	(2.79–5.922)	(3.60–15.30)	(179.71–424.08)	(46.35–594.96)	(138.48–249.46)	(99.04–214.85)	(21.36–65.68)	(2.58–6.04)
		**Score 0–1** (*n* = 20)	9.76	**1.53**	10.31	2.89	2.02	358.84	15.54	342.37	1050.95	78.85	8.84	158.50	5.81	**439.16**	4.39	5.17	215.29	379.61	142.59	120.50	33.00	2.94
			(5.58–23.39)	**(0.32–2.97)**	(6.60–13.48)	(0.46–3.46)	(0.69–3.51)	(209.52–507.16)	(7.64–23.53)	(273.40–406.18)	(698.11–2010.73)	(37.52–113.20)	(6.28–11.05)	(123.80–181.60)	(4.67–6.85)	**(364.36–519.80)**	(2.69–6.47)	(1.85–12.31)	(129.82–390.89)	(83.42–553.97)	(119.35–188.27)	(99.25–140.37)	(18.36–45.55)	(2.40–3.96)
		***p***	0.547	**0.042**	0.078	0.331	0.320	0.949	0.592	0.701	0.179	0.511	0.256	0.223	0.597	**0.022**	0.867	0.280	0.212	0.724	0.087	0.405	0.370	0.099
	**Men** (*n* =111)	**Score 2–3** (*n* = 73)	12.81	1.56	8.94	2.89	2.53	317.15	14.51	332.93	1445.90	55.94	8.67	158.90	5.30	**550.00**	5.94	8.02	341.06	391.61	**174.84**	138.56	38.57	3.16
			(5.30–36.03)	(0.34–3.16)	(5.67–13.20)	(0.30–3.56)	(1.06–4.20)	(219.38–422.57)	(14.51–24.35)	(248.81–583.79)	(999.83–2112.90)	(36.36–93.91)	(7.12–16.47)	(121.75–207.15)	(3.80–7.15)	**(464.11–636.44)**	(3.71–7.04)	(4.12–14.61)	(204.19–503.79)	(64.33–594.96)	**(137.98–210.82)**	(105.79–195.44)	(27.56–58.82)	(2.16–4.81)
		**Score 0–1** (*n* = 38)	6.37	0.99	8.26	3.21	2.04	310.36	13.61	315.29	1337.75	61.21	9.16	191.60	5.40	**459.15**	6.12	7.41	303.41	87.96	**150.12**	122.28	30.49	2.66
			(3.64–23.88)	(0.30–2.17)	(5.06–13.92)	(1.06–3.58)	(0.65–3.06)	(224.29–415.37)	(9.66–23.30)	(268.79–465.49)	(1024.98–1951.30)	(32.10–111.41)	(6.97–16.02)	(135.58–213.03)	(3.83–6.90)	**(411.93–565.90)**	(2.95–7.84)	(3.82–12.80)	(176.53–435.01)	(58.04–450.10)	**(120.12–181.45)**	(94.30–154.62)	(22.16–46.11)	(1.92–3.54)
		***p***	0.275	0.226	0.816	0.378	0.400	0.877	0.524	0.901	0.452	0.931	0.869	0.799	0.995	**0.012**	0.555	0.745	0.469	0.067	**0.023**	0.058	0.122	0.075

**Table 7:** Circulating biomarkers in relation to the presence of basal ganglia EPVS (bgEPVS) in women and men. Results are expressed as median (range).

			IL-4 [pg/mL]	IL-6 [pg/mL]	IL-8 [pg/m]	IL-10 [pg/m]	TNF-α [pg/mL]	CCL-2 [pg/mL]	CXCL-10 [pg/mL]	ICAM-1 [ng/mL]	VCAM-1[ng/mL]	VEGF [pg/mL]	PAI-1 [ng/mL]	vWF [%]	EMMPRIN [ng/mL]	MMP-2 [ng/mL]	MMP-7 [ng/mL]	MMP-8 [ng/mL]	MMP-9 [ng/mL]	MMP-12 [ng/mL]	TIMP-1 [ng/mL]	TIMP-2 [ng/mL]	TIMP-3 [ng/mL]	TIMP-4 [ng/mL]
**bgEPVS**	**Women** (*n* = 59)	**Present** (*n* = 30)	12.81	1.98	8.75	2.89	2.04	355.41	16.11	332.19	1621.6	60.43	8.93	175.60	5.79	526.44	4.15	6.78	318.51	**584.12**	169.12	**138.15**	38.82	4.02
			(5.30–32.61)	(1.49–5.40)	(5.44–13.32)	(0.61–3.46)	(0.73–4.50)	(262.32–571.88)	(10.39–21.91)	(264.93–507.81)	(1037.30–2294.40)	(38.53–118.09)	(5.74–13.85)	(129.20–210.70)	(4.35–7.78)	(398.94–668.58)	(2.58–5.53)	(3.97–12.06)	(170.94–409.15)	**(327.72–612.28)**	(148.52–236.37)	**(112.91–196.54)**	(25.72–55.20)	(2.72–6.37)
		**Absent** (*n* = 29)	12.81	1.94	5.98	3.23	2.28	322.89	16.96	359.47	1391.40	78.16	9.60	151.65	5.66	455.83	5.26	6.82	283.98	**283.50**	138.29	**116.54**	31.20	3.41
			(5.55–28.52)	(0.38–3.48)	(4.65–10.58)	(0.31–4.55)	(1.20–4.00)	(228.99–397.79)	(9.78–20.92)	(297.77–413.67)	(908.94–1997.23)	(46.81–114.64)	(6.87–13.44)	(131.15–202.78)	(4.25–6.87)	(375.42–677.35)	(2.87–7.14)	(1.71–14.76)	(154.55–419.03)	**(40.99–450.10)**	(118.62–208.78)	**(86.93–143.42)**	(15.88–53.65)	(2.35–4.79)
		***p***	0.798	0.538	0.172	0.387	0.905	0.120	0.854	0.374	0.429	0.638	0.698	0.655	0.861	0.228	0.151	0.719	0.743	**0.003**	0.092	**0.042**	0.384	0.136
	**Men** (*n* =111)	**Present** (*n* = 72)	12.81	**1.56**	9.33	3.21	2.53	326.68	13.49	328.62	1424.40	66.41	8.60	163.90	5.61	540.58	5.81	8.01	330.28	319.73	**173.42**	142.00	38.70	3.10
			(5.10–32.69)	**(0.38–3.51)**	(5.93–14.44)	(0.30–3.56)	(1.06–4.25)	(225.23–441.34)	(10.04–23.19)	(251.83–584.99)	(979.08–2147.70)	(37.15–111.16)	(7.15–16.51)	(126.60–213.00)	(4.37–7.61)	(435.16–627.28)	(3.26–7.13)	(3.90–14.06)	(216.41–483.71)	(76.16–542.51)	**(142.92–211.32)**	(109.98–192.599)	(28.68–56.69)	(2.29–5.04)
		**Absent** (*n* = 39)	6.50	**0.81**	7.12	2.89	2.03	279.09	15.42	314.39	1347.80	49.88	9.33	178.50	4.79	505.60	6.07	7.36	284.13	164.41	**154.32**	120.42	34.09	2.67
			(2.54–21.03)	**(0.30–1.89)**	(4.22–10.79)	(0.30–3.56)	(0.70–3.01)	(213.43–391.25)	(10.86–25.04)	(243.59–484.78)	(1037.43–1886.23)	(32.10–79.81)	(6.79–12.75)	(128.30–205.30)	(3.38–6.39)	(451.40–620.19)	(4.88–7.31)	(3.98–13.86)	(174.68–475.02)	(53.11–603.62)	**(114.72–194.56)**	(91.55–161.85)	(19.30–50.77)	(2.04–3.85)
		***p***	0.417	**0.019**	0.065	0.551	0.378	0.315	0.457	0.521	0.799	0.106	0.542	0.876	0.121	0.487	0.481	0.857	0.508	0.657	**0.018**	0.046	0.084	0.160

**Table 8:** Circulating biomarkers in relation to the presence of SVD markers according to the SVD score in women and men. Results are expressed as median (range).

			IL-4 [pg/mL]	IL-6 [pg/mL]	IL-8 [pg/mL]	IL-10 [pg/mL]	TNF-α [pg/mL]	CCL-2 [pg/mL]	CXCL-10 [pg/mL]	ICAM-1 [ng/mL]	VCAM-1 [ng/mL]	VEGF [pg/mL]	PAI-1 [ng/mL]	vWF [%]	EMMPRIN [ng/mL]	MMP-2 [ng/mL]	MMP-7 [ng/mL]	MMP-8 [ng/mL]	MMP-9 [ng/mL]	MMP-12 [ng/mL]	TIMP-1 [ng/mL]	TIMP-2 [ng/mL]	TIMP-3 [ng/mL]	TIMP-4 [ng/mL]
**SVDs**	**Women** (*n* = 59)	**At least one sign** (*n* = 28)	12.81	2.23	8.85	2.89	2.95	336.67	15.94	333.92	1667.15	78.69	9.47	**186.85**	5.75	556.20	3.92	7.63	309.33	**585.46**	**178.18**	**144.01**	**41.46**	**4.37**
			(5.73–34.21)	(1.49–5.52)	(4.77–15.22)	(0.30–3.46)	(1.07–5.77)	(256.53–533.83)	(10.52–21.06)	(269.41–532.19)	(1075.55–2345.95)	(46.21–120.91)	(6.55–12.65)	**(137.88–227.70)**	(3.95–8.08)	(448.54–739.46)	(2.54–5.66)	(3.53–15.73)	(167.33–404.75)	**(270.69–620.93)**	**(149.16–253.90)**	**(116.89–240.59)**	**(27.56–66.80)**	**(3.05–6.54)**
		**No signs** (*n* = 31)	12.81	1.73	8.0 5	2.89	2.03	341.05	16.37	342.99	1312.40	77.87	8.99	**146.65**	5.53	465.93	4.87	6.78	288.90	**309.12**	**138.48**	**114.31**	**30.49**	**2.89**
			(5.40–25.87)	(0.38–3.38	(4.65–10.62)	(0.44–3.56)	(1.10–3.51)	(227.89–455.04)	(8.61–22.99)	(282.06–408.06)	(888.10–2044.30)	(37.46–113.39)	(7.13–15.93)	**(126.00–198.20)**	(4.31–6.50)	(365.75–598.17)	(2.86–6.53)	(1.91–12.97)	(173.21–424.08)	**(45.01–463.50)**	**(112.96–202.86)**	**(84.31–136.99)**	**(15.73–46.61)**	**(2.31–4.02)**
		***p***	0.648	0.155	0.189	0.398	0.342	0.509	0.982	0.933	0.184	0.791	0.702	**0.049**	0.891	0.074	0.431	0.595	0.970	**0.015**	**0.011**	**0.003**	**0.032**	**0.009**
	**Men** (*n* =111)	**At least one sign** (*n* = 71)	12.81	1.56	9.33	2.89	2.53	317.86	14.34	328.62	1445.90	61.58	8.67	163.90	5.63	**556.06**	5.90	8.22	341.06	359.51	**177.31**	138.56	38.82	3.12
			(5.10–35.81)	(0.38–3.07)	(5.93–13.24)	(0.30–3.50)	(1.06–4.25)	(225.23–421.91)	(10.74–23.19)	(250.98–627.24)	(979.08–2147.70)	(37.46–108.71)	(7.15–16.42)	(124.60–211.00)	(4.13–7.61)	**(460.00–653.05)**	(3.70–7.11)	(4.00–14.76)	(220.49–549.24)	(62.96–594.96)	**(141.79–211.09)**	(106.63–196.96)	(28.59–58.76)	(2.14–5.40)
		**No signs** (*n* = 40)	6.50	0.81	7.77	3.20	2.04	309.25	14.15	317.89	1337.75	49.52	9.16	185.00	4.97	**468.36**	6.05	7.20	303.41	87.96	**150.12**	123.48	30.49	2.67
			(4.18–27.56)	(0.30–2.54)	(4.54–13.00)	(0.55–3.63)	(0.78–3.75)	(218.55–427.18)	(10.15–24.48)	(246.20–471.79)	(1034.68–1771.58)	(31.00–83.05)	(6.91–16.30)	(128.90–212.70)	(3.82–6.14)	**(424.51–557.19)**	(3.17–7.31)	(4.14–11.94)	(180.01–405.05)	(62.96–495.36)	**(115.48–175.69)**	(93.68–155.97)	(21.36–46.91)	(2.14–3.66)
		***p***	0.497	0.073	0.248	0.479	0.453	0.976	0.929	0.708	0.589	0.162	0.724	0.711	0.165	**0.014**	0.801	0.687	0.708	0.267	**0.009**	0.060	0.061	0.124

## Data Availability

The data presented in this study are available on request from the corresponding author. The data are not publicly available due to privacy or ethical restrictions.
